# A method for rapid demolition of a crossline arch bridge: a case study

**DOI:** 10.1038/s41598-024-53058-4

**Published:** 2024-02-05

**Authors:** Cixiang Zhu, Xiongjun He, Junyao Xiao

**Affiliations:** 1 Wuhan University of Technology, Wuhan, 430070 China; 2 CCCC Second Navigation Engineering Bureau Co., Ltd, Wuhan, 430000 China; 3 China Highway Engineering Consulting Group Co., Ltd, Beijing, 100089 China; 4 Xiamen University, Xiamen, 361005 China

**Keywords:** Civil engineering, Mechanical engineering

## Abstract

To reduce the adverse impact of demolition of the symmetrical rigid frame arch bridge overcrossing the highway with over-saturated traffic flow, a rapid demolition method based on the Self-Propelled Modular Transporter (SPMT) technique was developed in this study. The calculation formulae for reaction forces of the supporting brackets, as well as driving force and stability of SPMTs, were derived by analyzing the stability, synchronization, and influencing parameters of the cut bridge body—transport system. In addition, a monitoring system during the whole process was developed to ensure the demolition safety. An application of demolishing a crossline symmetrical rigid frame arch bridge in China within 5 h has been presented. The results showed that the proposed method can be successfully applied in real projects, leading to significant reduction in traffic impact, energy consumption, and environmental pollution.

## Introduction

With the rapid development of China’s economy, the operational traffic volume of some expressways has reached a state of severe overcapacity. There is an urgent need to expand and upgrade these expressways by adding 2–6 lanes on one or both sides of the existing bidirectional 4 to 6-lane expressways in order to accommodate the growing traffic demand^1–4^. However, this expansion necessitated the demolition of the bridges crossing over the expressway within critical networks. Given the bridge’s design, the high volume of traffic flowing beneath it, the complex environment of the demolition project, and other challenges, demolishing such bridges poses significant difficulties and elevated technical risks^5,6^. The demolition technology used for existing bridges directly impacts the safety of expressway operations.

Conventional methods for demolishing bridges primarily encompass static cutting, mechanical chiseling, and blasting control^7–10^, etc. During the bridge demolishing process, these methods usually encounter certain issues, such as low efficiency, high construction risks, and significant traffic disruptions^11–14^. Particularly when dealing with lightweight structures like symmetrical rigid frame arch bridges, limited integrity, and horizontal thrust, the demolition process demands a full expressway closure lasting between 6 and 120 h. This scenario presents practical challenges like heightened risks and substantial traffic disturbances. As a result, further research into rapid demolition techniques for crossline symmetrical rigid frame arch bridges becomes essential. This is to ensure optimal construction safety and minimize traffic disruptions as much as possible.

The US Federal Highway Administration has introduced the Accelerated Bridge Construction (ABC) method as a means to enhance construction speed, shorten overall construction duration, and mitigate both traffic disturbances and project costs^15–19^. They have also successfully carried out the rapid demolition and replacement of more than 100 bridge superstructures, with notable results based on the SPMT construction method. The Self-Propelled Modular Transporter (SPMT) was firstly used in ABC approach. Utilizing the SPMTs, the complex sequence of on-site bridge superstructure construction can be streamlined into an efficient step: the relocation of the prefabricated bridge superstructure directly to its designated position. Then this method has gained substantial popularity in the retrofitting of aged bridges, primarily due to its ability to significantly reduce traffic interference, diminish environmental footprint, enhance overall quality, and decrease the total cycle costs. In China, Beijing Municipal Group and Qin Dahang et al.^18,20^ integrated the development of a 1000-ton special SPMT workhorse, which was successfully applied to the rapid replacement of the superstructure of Xiguan Ring Island Bridge and Sanyuan Bridge in Beijing.

On the basis of previous work^21,22^, this study has developed a non-blasting, efficient rapid demolition technology for crossline symmetrical rigid frame arch bridge based on SPMT, the aim of which is to minimize traffic closure duration and mitigate the environmental and traffic impact during the demolition process. The investigation focuses on addressing the critical technical challenges encountered during the demolition procedure. A comprehensive analysis of stability, synchronization, and influential parameters of the cut bridge body—transport system has led to the derivation of formulae for calculating reaction forces of the supporting brackets, as well as driving forces and stability of the SPMTs. Additionally, to ensure the safety of the demolition process, a comprehensive monitoring system has been integrated to oversee the entire operation. This added layer of monitoring provides real-time insights and guarantees the well-being of the procedure. Illustrating the efficacy of the proposed approach, an application showcases the successful demolition of a crossline symmetrical rigid frame arch bridge in China, accomplished within a remarkable 5-h timeframe. This real-world application underscores the feasibility of the method in practical scenarios. Noteworthy outcomes include substantial reductions in traffic disturbances, energy consumption, and environmental pollutants.

## SPMT-based rapid demolition method

Addressing the unique challenges posed by the high traffic volume and complex surroundings of the expressway, an SPMT-based rapid demolition method for the symmetrical rigid frame arch bridge crossing over the expressway has been proposed in this study. The primary objective of this approach is to ensure the demolition operations can be conducted with due regard for safety, operational efficiency, and minimal traffic disruption.

### Workflow of the SPMT-based rapid demolition method

The procedural workflow encompasses the following key phases:

1. On-site preparations:

Initial on-site preparations mainly include leveling and hardening the temporary storage site, setting up the enlarged foundation, fabricating the carrying brackets, and removing the central divider on the temporary transit route.

(2) Pre-cutting of bridge body:

Before the closure of traffic, the side spans and diagonal braces of the rigid frame arch bridge are symmetrically cut, hoisted, and moved in discrete blocks to reduce the traffic closure duration. The cutting positions are determined by the preliminary Finite Element (FE) analysis.

(3) Assembly of the transport system:

The transport system includes the support brackets and several SPMTs. The support brackets are composed of steel pipe columns, connecting rods, and distribution beams. The SPMTs are arranged according to the direction of the bridge and the sup-porting force can be determined by the formulae proposed in the study.

(4) Pre-lifting of the transport system:

With the traffic fully closed and the temporary transit route cleared of obstructions, the transport system is meticulously positioned beneath the existing bridge. The lifting height of each SPMT is judiciously adjusted to ensure the supporting bracket is close to the bottom of the bridge. Then the lifting load is incrementally applied according to the self-weight of the cut bridge body.

(5) Precise cutting of bridge body:

A synchronized chain-saw arrangement is deployed along each arch leg of the rigid frame arch bridge, enabling the cutting to be fast and simultaneous. The cutting posi-tions are also determined by the FE analysis.

(6) Movement of the cut bridge body:

The cut bridge body is lifted synchronously by approximately 20 cm, and then moved by the transport system along the temporary transit route to the temporary storage site, after which the traffic can be resumed. The driving forces and the stability of SPMTs are determined by the formulae proposed in this study.

Among these phases, there are two important technical issues to be addressed. First of all, the reaction forces of the supporting brackets should be determined to avoid buckling and collapse of the columns in the supporting brackets. Then, the driving forces and stability of the SPMTS should be examined to ensure the SPMTs can carry and move the cut bridge body smoothly.

### Determination of reaction forces of the supporting brackets

**Figure 1:**
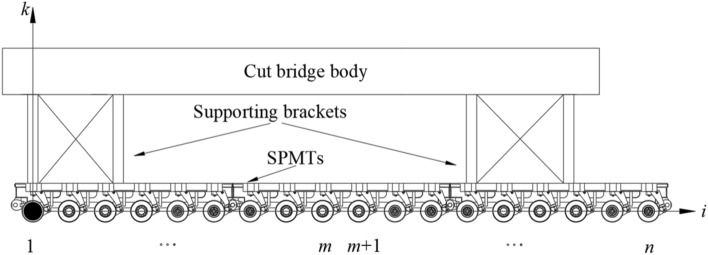
Elevation of cut bridge body, supporting brackets and SPMTs (*n* rows × *v* columns).

**Figure 2:**
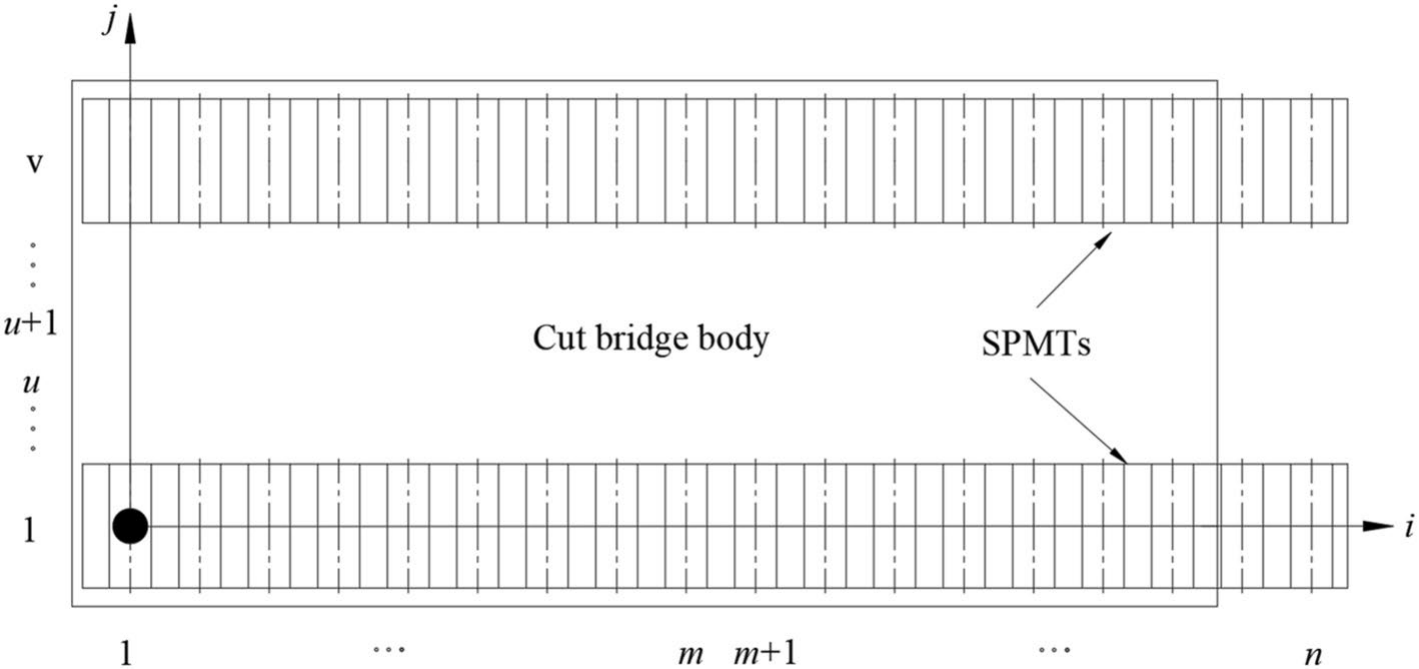
Top view of cut bridge body, supporting brackets and SPMTs (*n* rows × *v* columns).

**Figure 3:**
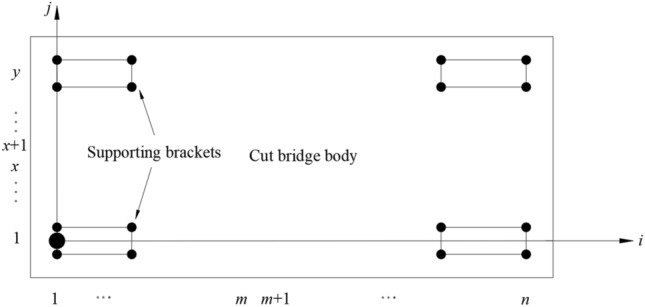
Top view of cut bridge body and supporting columns.

**Figure 4:**
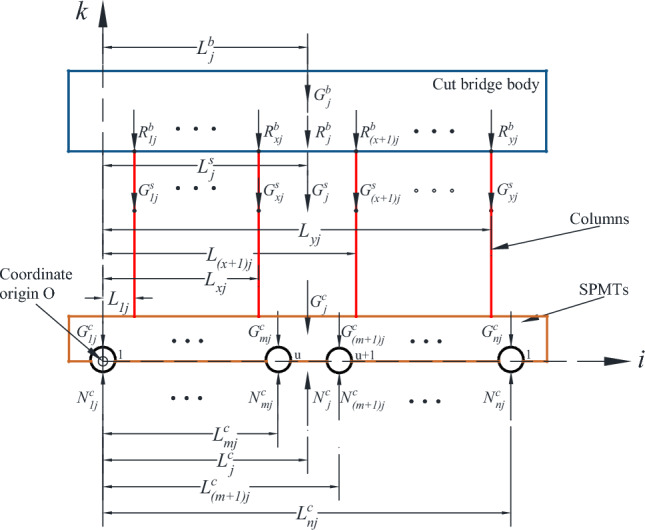
Free body diagram of cut bridge body, supporting brackets and SPMTs in the jth column.

With the center of the first wheel axle in the first column module group as the co-ordinate origin, the longitudinal direction of the displaced bridge section as the i axis, the transverse direction of the bridge as the j axis, and the longitudinal direction of the bridge as the k axis, a three-dimensional rectangular coordinate system was estab-lished, as shown in Figs. 1, 2 and 3. Figure 4 is the basic force system diagram of the cut bridge body, supporting brackets and SPMTs in the jth column.

The equilibrium equation for the SPMTs in the jth column can be written as

1 $$ N_{j}^{c} = R_{j}^{b} + G_{j}^{s} + G_{j}^{c} \le \left[ {S_{j}^{c} } \right] = S_{j}^{c} /k_{j}^{c} $$

where ${N}_{j}^{c}$ is the total road reaction force to the axle suspension of the SPMTs in the *j*th column, ${R}_{j}^{b}$ is the total force of the cut bridge body on the *j*th support column, ${G}_{j}^{s}$ is the total weight of the supporting columns carried by the SPMTs in the *j*th column, including the total weight of the columns and the total weight of the connecting structures between the columns, ${G}_{j}^{c}$ is the total self-weight of the vehicle body for the module group in the* j*th column, $[{S}_{j}^{c}]$ is the allowable load of the double suspension of all the SPMTs in the *j*th column, ${S}_{j}^{c}$ is the maximum load capacity of the *j*th column SPMTs, ${k}_{j}^{c}$ is the load safety factor of the *j*th column SPMTs.

The equilibrium equation of static moment of each vertical force acting on the column in the brackets can be obtained as

2 $$ \mathop \sum \limits_{{\text{i = 1}}}^{n} \left( {R_{{{\text{ij}}}}^{b} *L_{ij} } \right) + \mathop \sum \limits_{{\text{i = 1}}}^{n} \left( {G_{{{\text{ij}}}}^{S} *L_{ij} } \right) + \mathop \sum \limits_{{\text{i = 1}}}^{v} \left( {G_{{{\text{ij}}}}^{c} *L_{ij}^{c} } \right) = \mathop \sum \limits_{{\text{i = 1}}}^{v} \left( {N_{{{\text{ij}}}}^{c} *L_{ij}^{c} } \right) $$

where ${L}_{ij}$ is the distance from $G_{ij}^{{\text{s}}}$ to the coordinate origin, ${L}_{ij}^{c}$ is the distance from ${N}_{ij}^{c}$ to the coordinate origin.

Then the difference between the resultant force of the bearing points composed of (*u* + 1) to the axle of *v* and the resultant force of the bearing points composed of 1 to the axle of *u* is

3 $$ \mathop \sum \limits_{i = u + 1}^{v} N_{{{\text{ij}}}}^{c} - \mathop \sum \limits_{i = 1}^{u} N_{{{\text{ij}}}}^{c} = 15.6*\left[ {\left( {p + n} \right)*\left( {v - u} \right) - pu} \right] $$

where *n* is the wheel pressure difference between the average oil pressure of the (*u* + 1)th to *v*th axle the average oil pressure p of the 1st to the *u*th axle.

Finally, the equilibrium equation of the cut bridge body and the transport system can be obtained as

4 $$ R^{b} = \mathop \sum \limits_{{\text{i = 1}}}^{y} R_{j}^{b} = \mathop \sum \limits_{{\text{i = 1}}}^{y} G_{j}^{b} = G^{b} $$

where ${R}^{b}$ is the total weight of the cut bridge body, ${R}_{j}^{b}$ is the total compression force of the *j*th supporting column, ${G}^{b}$ is the sum of the local bridge body weight, and ${G}_{j}^{b}$ is the local bridge body weight suffered by the *j*th supporting column.

### Determination of driving force and stability of the SPMTs

Based on Eqs. (1–4), the reaction force of the SPMTs in each column can be determined. It should be noted that the different pressure between wheels is important, which is affected by the relative height difference of the SPMTs and the roughness of temporary transit route. In the case study, the maximum allowable wheel pressure difference at the front and rear support points was set to 10 MPa. By using this calculation model, the reaction force of the SPMTs can be evaluated more accurately, and the safety and stability of bridge in the process of rapid transportation can be ensured.

Subsequently, the driving force of the SPMTs (${F}_{D}$) can be written as

5 $$ F_{D} = N_{j}^{c} \mu + N_{j}^{c} \sin (\arctan \gamma ) \le F_{k} /k_{f} $$

where $\mu$ is the friction coefficient between ground and SPMT wheel, $\gamma$ is the slope of the transit route, ${F}_{k}$ is the maximum driving force and ${k}_{f}$ is the safety coefficient.

**Figure 5:**
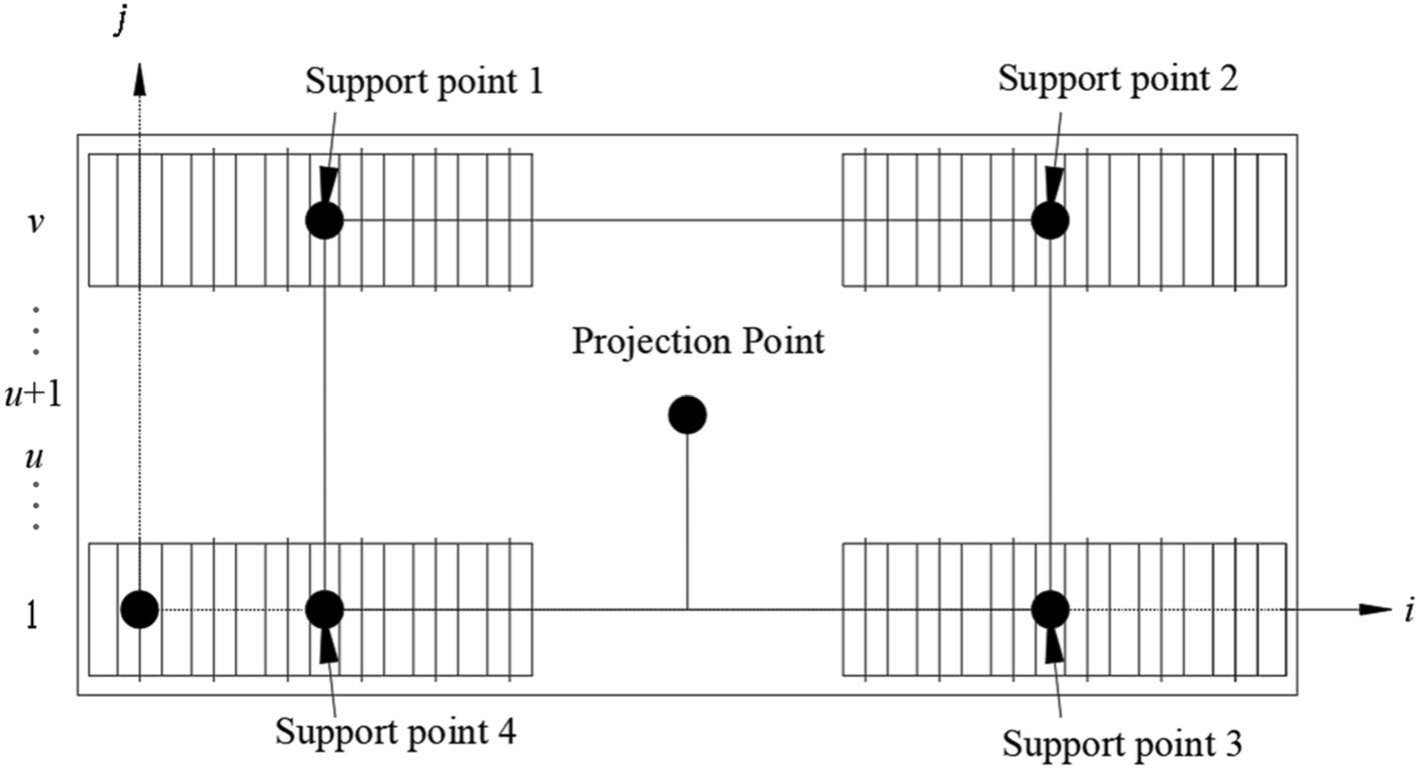
Diagram of four point support by the SPMTs.

**Figure 6:**
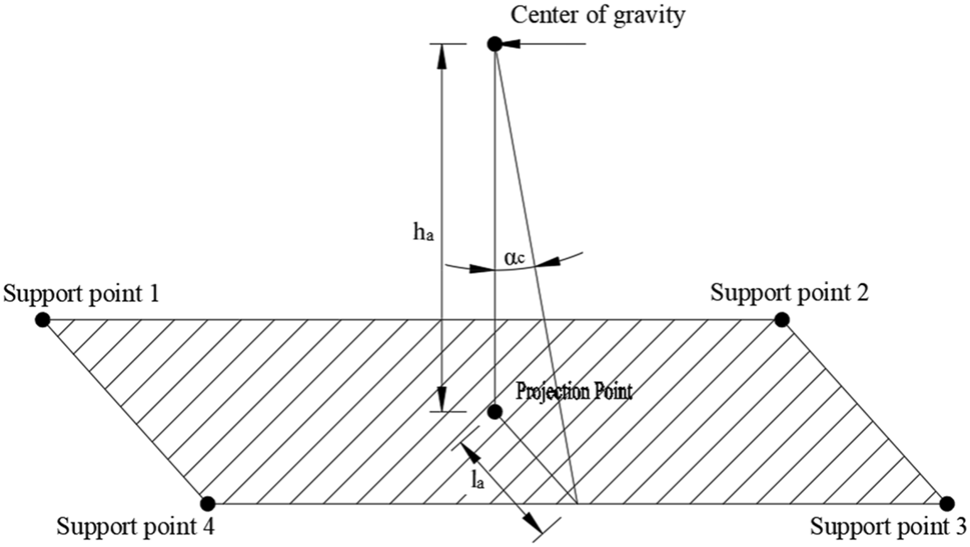
Schematic diagram of calculating the transportation stability angle for the cut bridge body under four-point support conditions.

During the transportation of the cut bridge body, it may have complex stress distribution, leading to the potential occurrence of local cracking^23,24^. Therefore, it is necessary to analyze the integrity and stability of the cut bridge body during the entire transportation. The diagram of four point support by the SPMTs is shown in Fig. 5. Based on the geometrical relationship, the stable angle can be calculated as:

6 $$ \alpha_{c} = \arctan \left( {\frac{{l_{a} }}{{h_{a} }}} \right) < \alpha_{x} $$

where ${\alpha }_{c}$ is transportation stability angle, ${l}_{a}$ is the minimum distance between the projection point of the total center of gravity of the transported bridge body and the combined support point on the SPMTs, ${h}_{a}$ is the distance between the total center of gravity of the transported bridge body and the combined support point on the SPMT vehicle, and ${\alpha }_{x}$ is the transport stability angle which can be considered as 7°. The calculation diagram of the stability angle is shown in Fig. 6. It is noteworthy that Eq. (6) can be used for different scenarios: the initial state (SPMT height of 1.2 m), normal transportation state (SPMT height of 1.5 m), and lifted state (SPMT height of 1.7 m).

## Application of the SPMT-based rapid demolition method

### Project information

**Figure 7:**
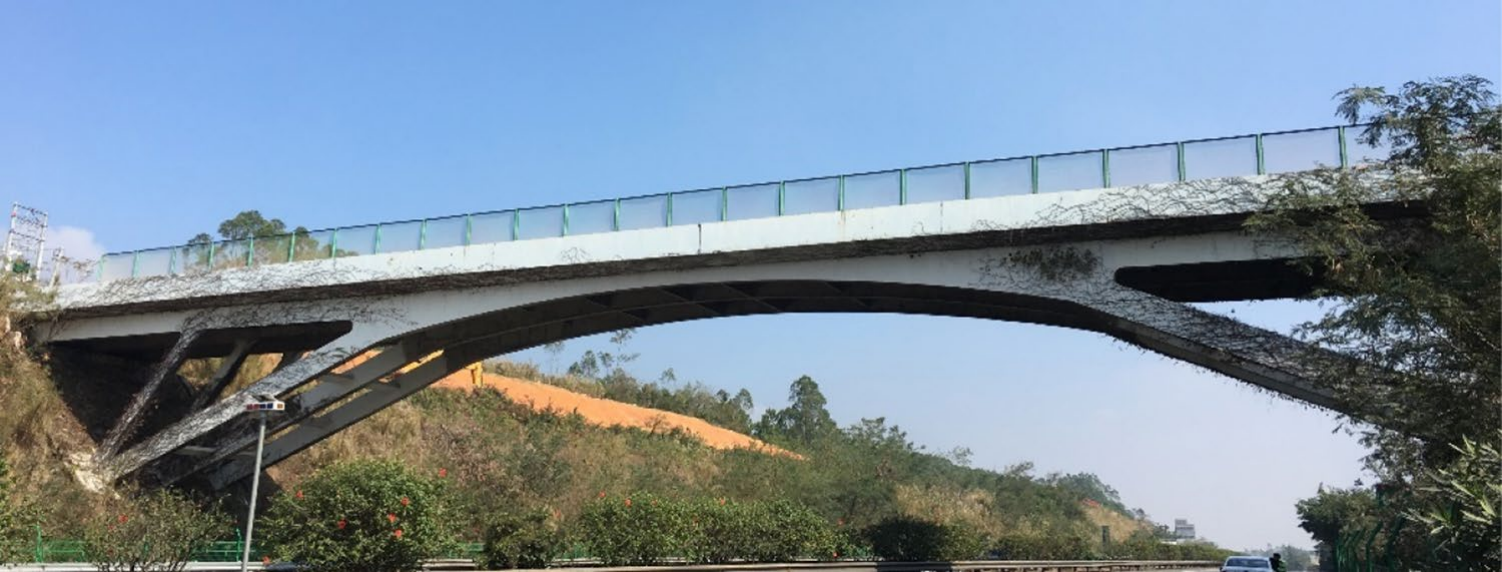
Photograph of the rigid-frame arch across the highway.

The expansion project of the Shenhai Expressway from Shuikou to Baisha in Guangdong Province, China, aimed to expand the existing dual four-lane road into a dual eight-lane road using a dual-side joint widening method. It increased the road width from 28 to 42 m. As part of this project, one rigid frame arch bridge crossing over the expressway needed to be dismantled and reconstructed, as shown in Fig. 7.

**Figure 8:**
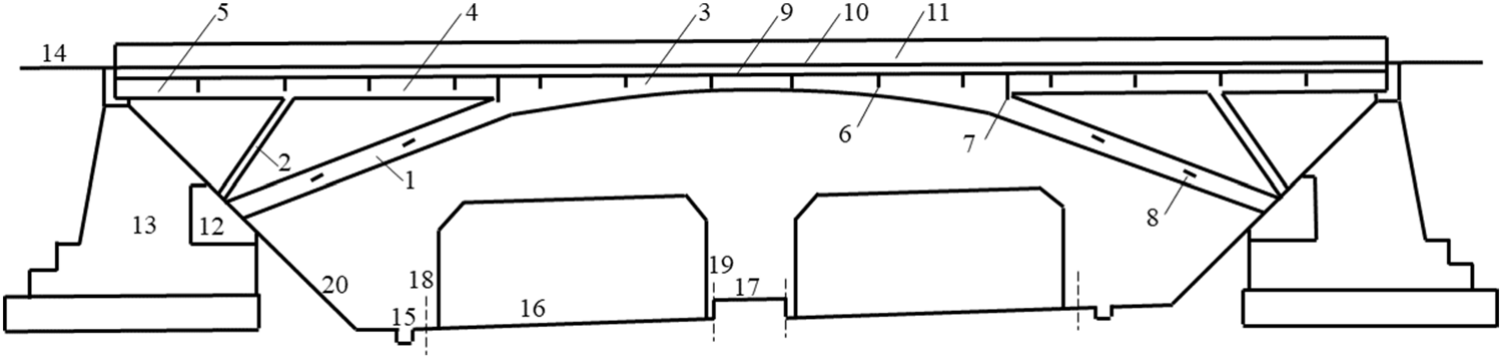
Elevation of rigid frame arch bridge across the highway.

Figure 8 shows the elevation of the rigid frame arch bridge. The upper structure of this rigid frame arch bridge consisted of rigid frame arches, transverse beams, and bridge decks. The bridge deck had a length of 61.6 m and a width of 8 m, while the rigid frame arches were composed of three sections of ribbed beams with heights ranging from 0.78 to 1.13 m. The maximum net height under the bridge was 9.55 m.

Due to the presence of horizontal loads, it is not suitable to close one side while keeping the other side open during demolition. On the other hand, conventional approaches like setting up protective scaffolding and compressing the lanes for bidirectional traffic can easily lead to traffic congestion and safety incidents. A more suitable approach during demolition would be the complete closure of the expressway. However, the traffic flow of the expressway can be up to 70,000 vehicles per day, so it cannot be closed for a long period of time. Therefore, it is necessary to develop a rapid demolition method so that the closure of expressway can be limited to within a few hours to reduce the adverse impact on the traffic.

### FE analysis

**Figure 9:**
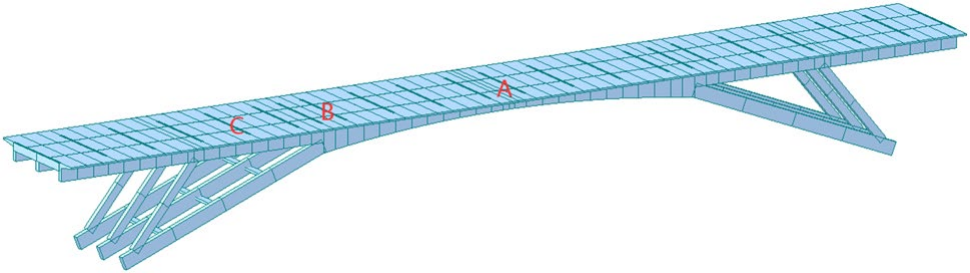
Schematic diagram of the FE model of the rigid frame arch bridge.

**Figure 10:**
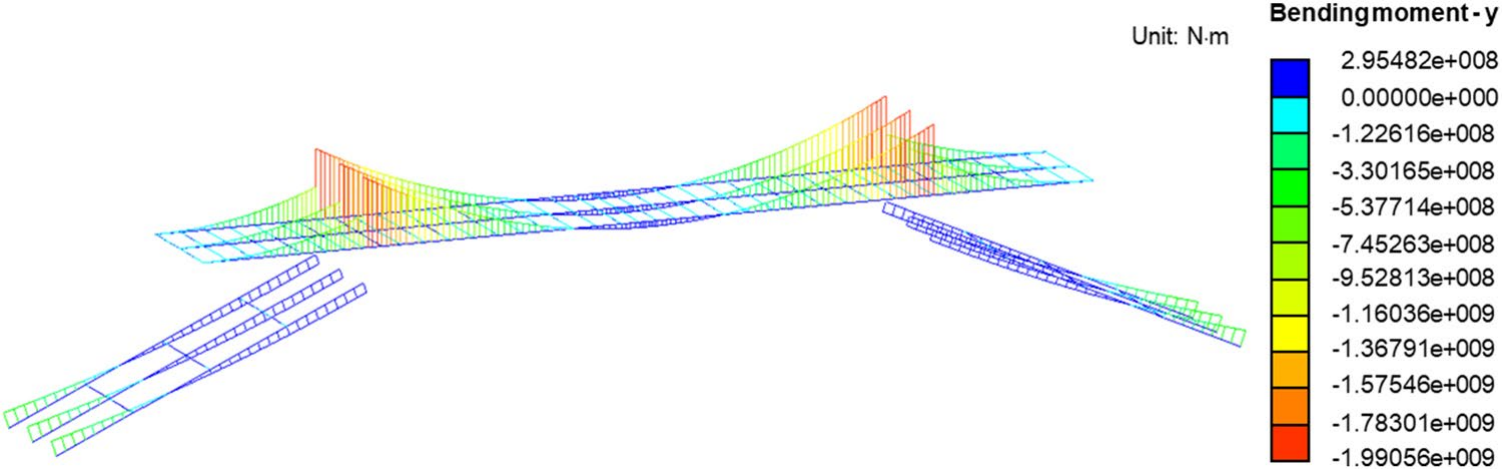
Bending moment diagram in Scenario 3.

**Figure 11:**
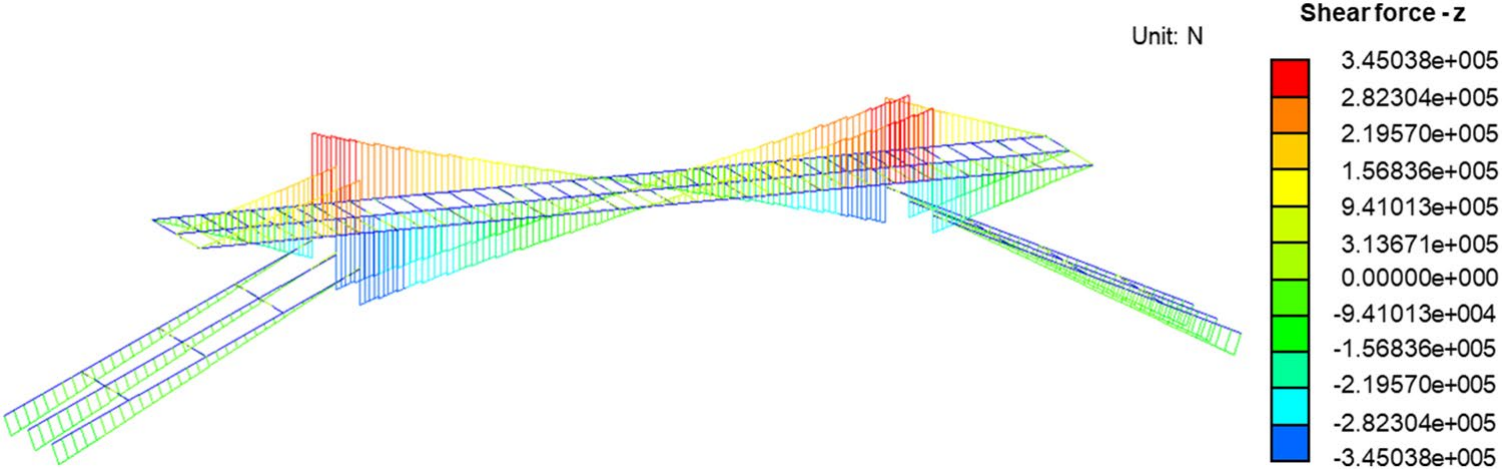
Shear force diagram in Scenario 3.

A FE model was established by MIDAS according to the actual dimensions of the rigid frame arch bridge, as shown in Fig. 9. The principal beam was composed of C30 grade concrete, exhibiting an elastic modulus of 3.3 × 10^4^ MPa, a Poisson’s ratio of 0.167, and a unit weight of 26 kN/m^3^. At the axial center, the concrete demonstrated a compressive strength of 28 MPa and a tensile strength of 2.6 MPa. The steel reinforcement, of HRB335 grade, exhibited an elastic modulus of 2 × 10^6^ MPa, a unit weight of 78.5 kN/m^3^, and a tensile strength of 340 MPa. During the demolition process, several scenarios were considered:

1. the bridge suffered the permanent load only;
2. the main span suffered the permanent load while the side spans were cut off;
3. the main span suffered the permanent load while the diagonal braces were cut-off;
4. the main span suffered the permanent load while the arch legs were cut off;

For the results of the numerical simulation, Fig. 10 depicts the diagram of the four-point support provided by the SPMTs, while Fig. 11 illustrates the stable angle of this four-point support. Due to space limitations, only the bending moment and shear force diagrams for Scenario 3 are presented in Figs. 10 and 11. In addition, Table 1 shows a comparison of the internal forces at different sections. From the calculation results, it is evident that the calculated bending moment and shear force values at critical sections are significantly lower than the theoretical resistance values after the cutting. Specifically, there is a clear negative bending moment at Cross-section B after cutting off the diagonal braces, and an increase in shear force is observed. Therefore, during the demolition process, measures should be taken to prevent concrete cracking and cantilever end failure at this cross-section.

**Table 1:** Sectional internal forces for different scenarios.

Position	Internal forces	Scenario 1		Scenario 2		Scenario 3		Scenario 4	
		FE	Limit	FE	Limit	FE	Limit	FE	Limit
Section A	M/kN m	259.18	1747	257.72	1747	− 235.56	1747	− 1589.34	2358
	V/kN	6.41	359	6.29	359	5.73	359	256.89	857
Section B	M/kN m	− 58.89	2358	− 44.631	2358	− 960.94	2358	− 355.75	7186
	V/kN	101.6	857	111.46	857	255.87	857	− 365.26	502
Section C	M/kN m	11.84	2470	− 62.43	2470	− 83.11	2470	167.05	1747
	V/kN	− 76.32	507	64.03	507	73.88	507	0.18	359

### Construction work

**Figure 12:**
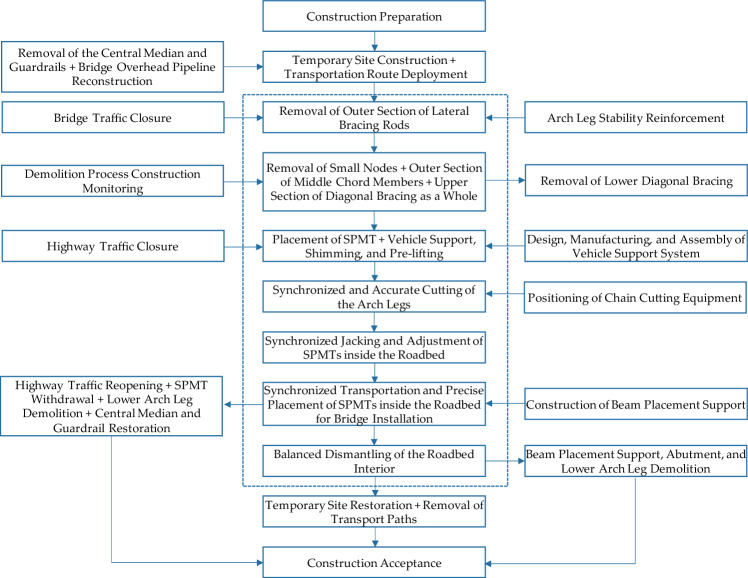
Flowchart of the overall work process of rapid demolition of rigid frame arch bridge.

Figure 12 shows the flowchart of the overall work process of rapid demolition of the rigid frame arch bridge. To minimize the impact on the expressway traffic and facilitate the organized construction, the upper structure of the rigid frame arch bridge was divided into two parts: the “inner bridge” and the “outer bridge”. The “inner bridge” included the inner segment of the main chord, the upper segment of the arch leg, the major node, the solid belly segment, and their corresponding transverse beams and bridge deck systems. On the other hand, the “outer bridge” comprised the outer segment of the main chord, the outer diagonal brace, the diagonal brace, the minor node, the lower segment of the arch leg, and their corresponding transverse beams and bridge deck systems.

**Figure 13:**
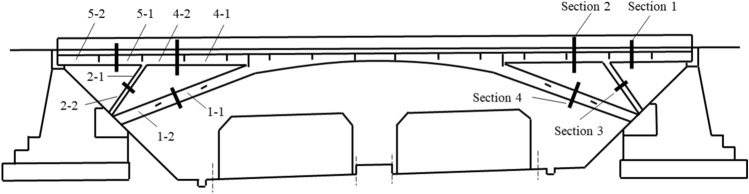
Cutting diagram of crossline rigid frame arch bridge.

**Figure 14:**
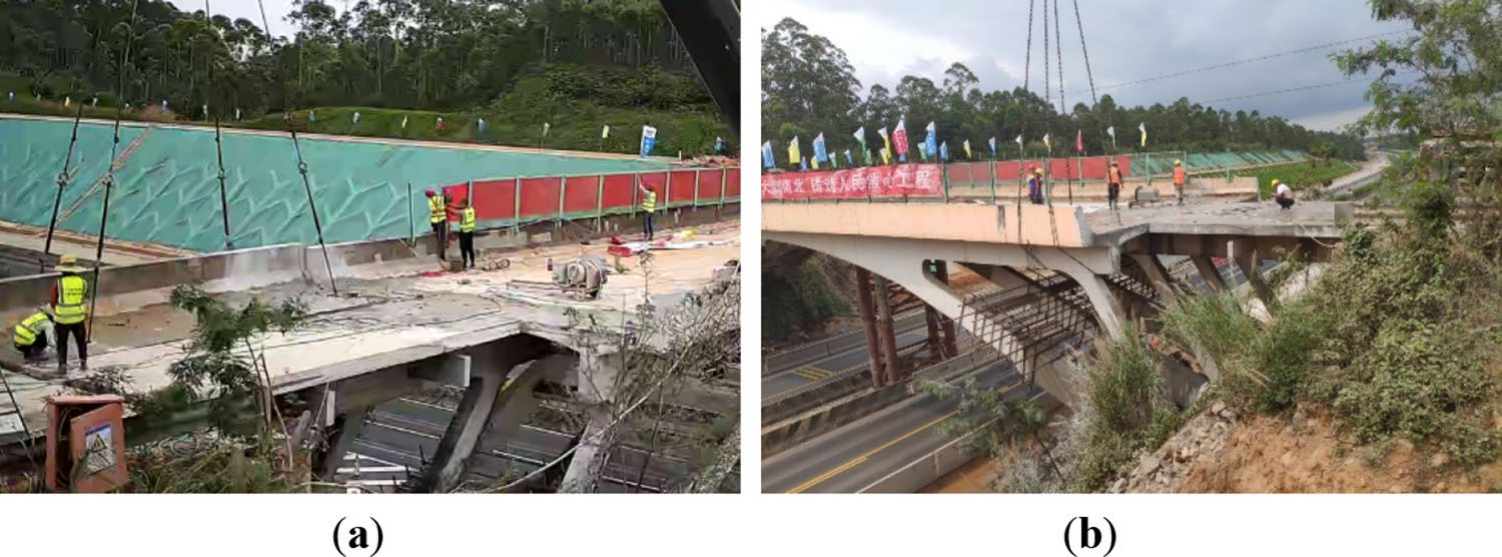
Hoisting of pre-cut bridge segments (**a**) outer segment 5–2 (**b**) inner segment 5–1.

After conducting a survey and assessment of the technical condition and boundary conditions of the rigid frame arch bridge, it was decided to demolish the “outer bridge” first (excluding the lower segment of the arch leg) and then proceed with the demolition of the “inner bridge”. More specifically, the cutting of the arch leg was divided into “upper segment 1–1” and “lower segment 1–2”, while the cutting of the diagonal brace was divided into “upper segment 2–1” and “lower segment 2–2”. Additionally, the cutting of the main chord was divided into “inner segment 4–1” and “outer segment 4–2”, and the cutting of the side chord was divided into “inner segment 5–1” and “outer segment 5–2”, as depicted in Fig. 13. Figure 14a,b show the hoisting of the pre-cut bridge segments, “outer segment 5–2” and “inner segment 5–1”, respectively.

Although the “outer bridge” was demolished before the “inner bridge” in this study, it is noteworthy that the sequence of demolishing the “inner bridge” and the “outer bridge” should be determined based on the construction requirements. If it is decided to demolish the “inner bridge” first or pre-cut the main chord into inner and outer segments, favorable time and space conditions for the rapid demolition of the “inner bridge” will be created. If the overall load-bearing capacity of the arch ribs (arch legs and solid belly segments) meets the safety requirements, demolition can be carried out without additional support. However, if the safety requirements are not met, appropriate structural support and safety measures need to be implemented, making it preferable to demolish the “inner bridge” before proceeding with the demolition of the “outer bridge”.

**Figure 15:**
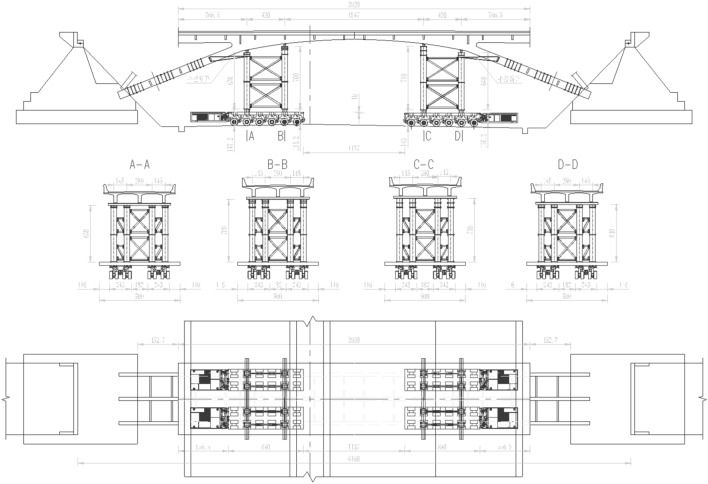
Arrangement of transport system including supporting brackets and SPMTs.

**Figure 16:**
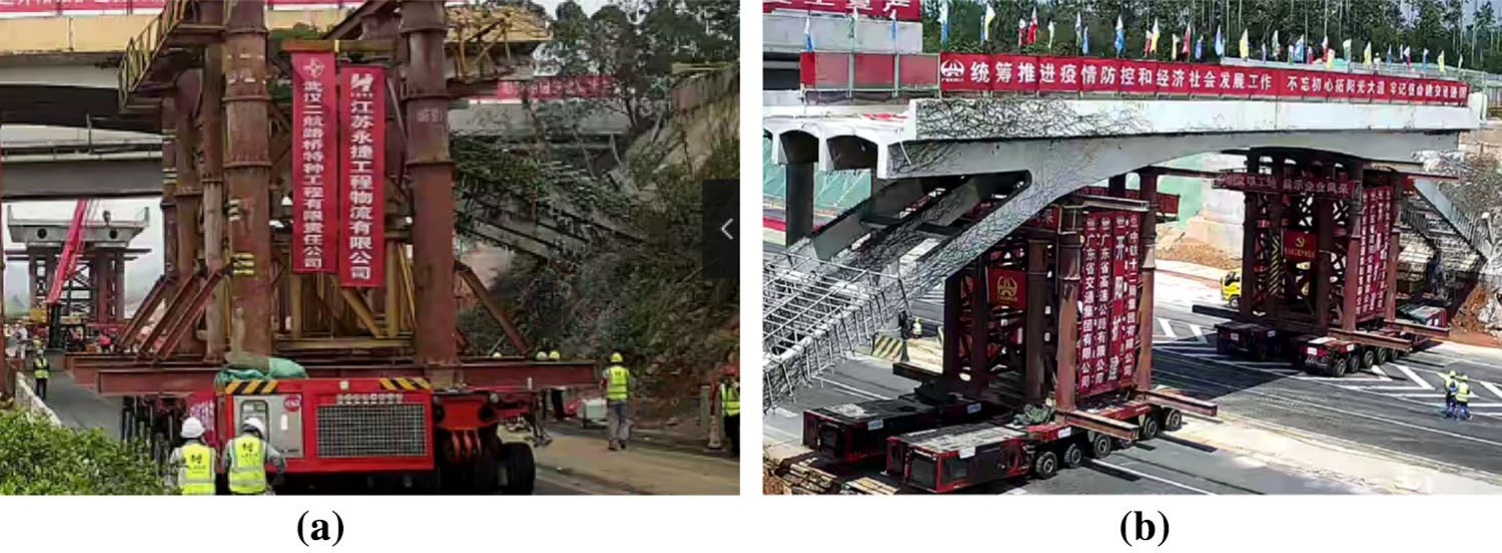
Photograph of (**a**) assembly of the transport system (**b**) pre-lifting of the transport system.

The weight of the rigid-frame arch bridge was approximately 480 t, and the minimum width of the on-site road was only 11.75 m. Therefore, 4 sets of independent 6-axle line SPMTs were employed to ensure the safe and efficient transportation of the bridge deck. The design plan for the rapid movement of the cut bridge body using SPMTs is shown in Fig. 15. Figure 16a,b show the assembly of the transport system and pre-lifting of the transport system, respectively.

**Figure 17:**
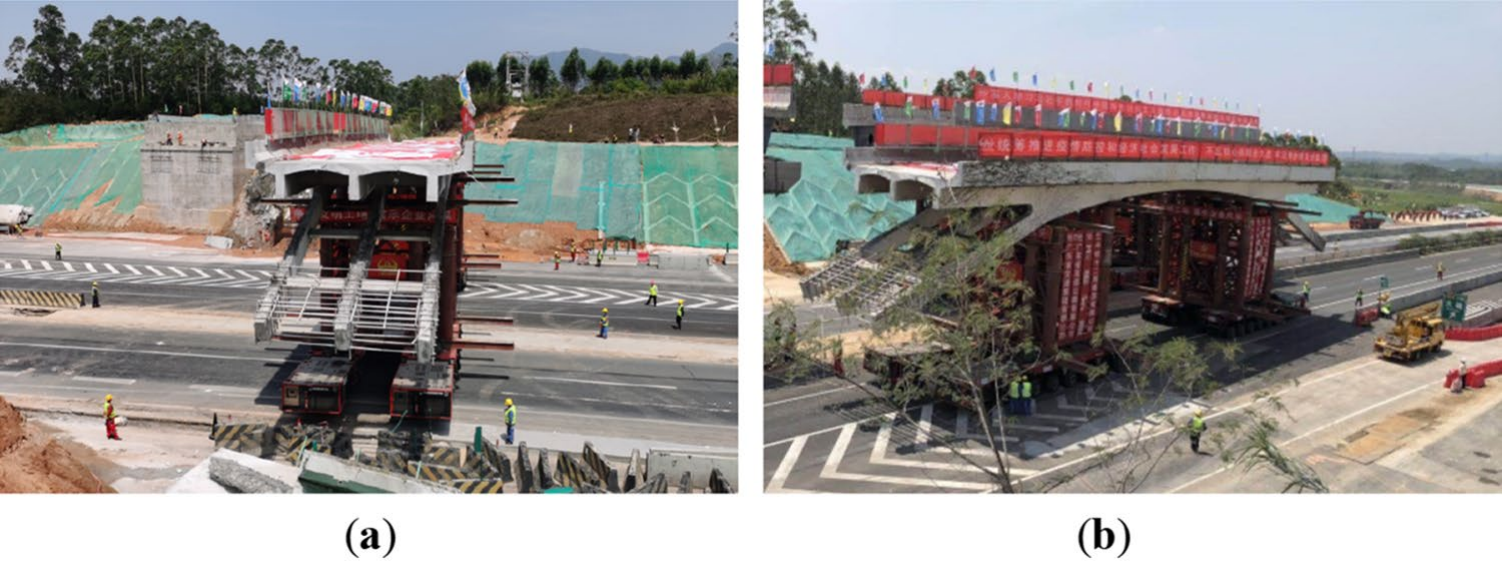
Movement of the cut bridge body (**a**) transversely movement (**b**) longitudinal movement.

**Figure 18:**
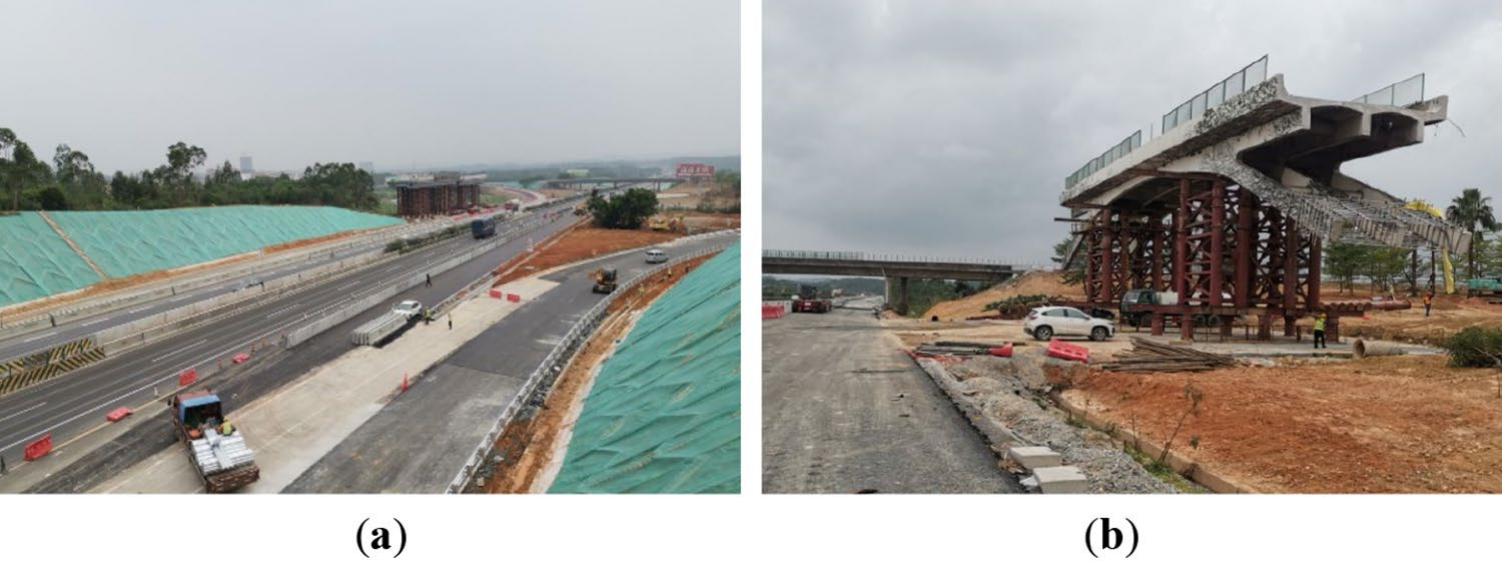
Photograph of (**a**) temporary transit route (**b**) temporary storage area.

During the movement of the cut bridge body, the transport system initially moved along the road for a certain distance while the cut bridge body was perpendicular to the expressway, as shown in Fig. 17a. Then the transport system underwent a 90° rotation on the site plane, changing from occupying both directions to a single direction lane, as shown in Fig. 17b. Along the temporary transit route shown in Fig. 18a, the cut bridge body finally arrived at the temporary storage area, which can be seen in Fig. 18b.

## Monitoring during the demolition process

To ensure the safety of the demolition of the rigid-frame arch bridge, monitoring was conducted during the construction work. Considering the longitudinal and transverse slopes on the expressway, as well as the potential safety risks during the turning, rotation, and transportation, sensors and testing instruments were pre-installed at critical locations of the bridge. A wireless monitoring network was then established using wireless intelligent sensor nodes, and real-time collected data, including geometric and mechanical parameters, were uploaded to an online monitoring cloud platform through a mature operator network (4G) using Internet of Things (IoT) transmission technology. Back-end computer programs analyzed and processed this data, enabling real-time monitoring and integration of on-site data.

**Figure 19:**
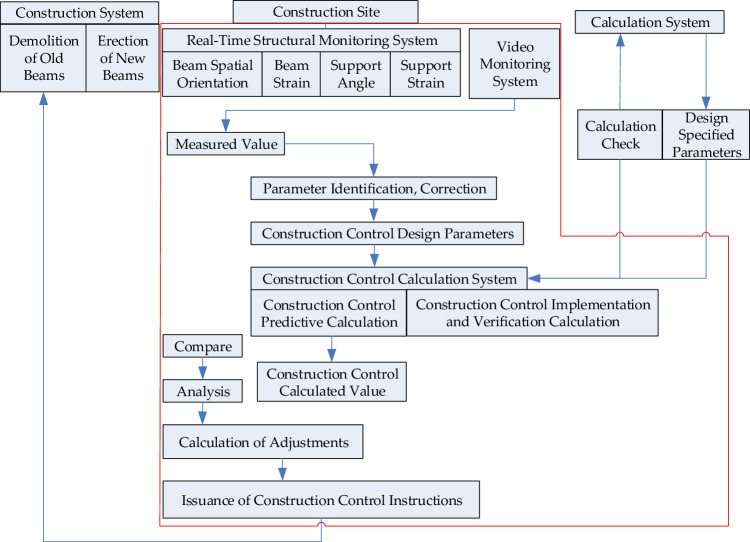
Flowchart of the monitoring during demolition.

Real-time dynamic monitoring was conducted to obtain real-time data on beam posture, relative displacement, and stress variations. The data could ensure that the structural forces and deformations remain within a safe and controllable range. It could also verify the calculated values against the actual conditions to ensure their consistency with the bridge’s real state. Additionally, the monitoring system had an “early warning” function, allowing timely detection of unsafe conditions during construction work and enabling necessary measures to be taken to ensure construction safety. Through this comprehensive monitoring and warning mechanism, construction risks were minimized, and the construction of the rigid-frame arch bridge over the highway was carried out safely and efficiently. The implementation system of the construction monitoring work is shown in Fig. 19.

**Figure 20:**
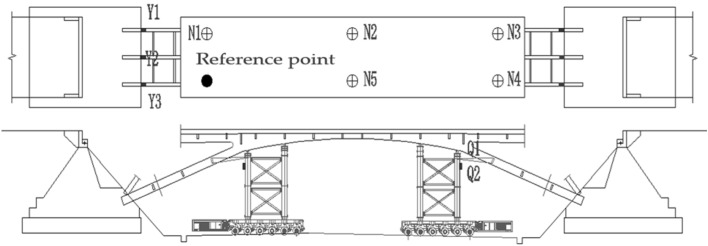
The layout of the sensors.

Six dynamic strain gauges (Y) were installed at the bridge abutments on both sides to monitor the stress changes before and after the cutting of the arch legs. Four inclinometers (Q) were installed at the outer four corners of the beam-carrying support to monitor the changes in the inclination of the support. Moreover, five precision level gauges (N) and a reference point (water tank) were placed on the bridge deck to monitor the spatial posture changes of the bridge body during the movement. The positions of the monitoring points are shown in Fig. 20.

**Figure 21:**
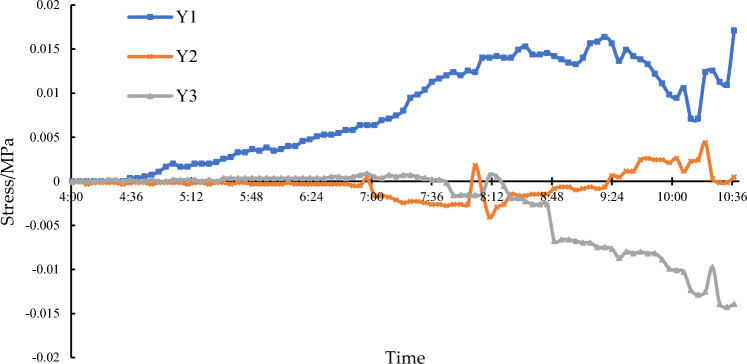
Monitoring of strain at arch feet.

At 7:00, the traffic on the expressway was closed. The stress at the arch feet is shown in Fig. 21. During the pre-cutting (4:00 to 7:00) and the arch leg chainsaw cutting (7:00 to 10:00), real-time strain monitoring showed minimal variation in stress at arch feet, with values fluctuating within a range of 0.02 MPa. This indicates that the main span structure remained stable during the cutting process. It is important to consider that concrete strain measurements can be influenced by various factors, including elastic strain caused by loads, shrinkage, creep, and temperature effects.

**Figure 22:**
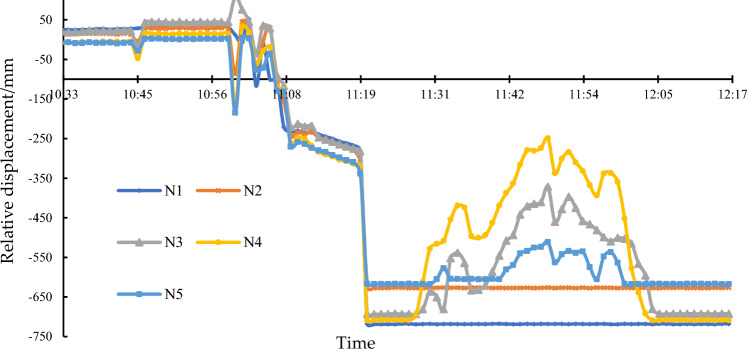
Monitoring of relative displacements on the bridge top surface.

To monitor the stability and structural safety of the cut bridge body during the transportation along the temporary transit route, real-time monitoring of vertical displacements was conducted, as shown in Fig. 22. It ensured that the cut bridge body remained stable and avoided potential risks such as slipping, overturning due to local support system failure, or damage caused by excessive stress at certain sections. Liquid static leveling instruments, including one benchmark point, were installed on the top surface of the cut bridge body. The measurement resolution was 1.0 mm.

After completing the arch leg cutting (at 10:00), the cut bridge body was supported by the transport system, and it converted to a simply supported system. The relative displacements at various monitoring points were all within the range of ± 50 mm, and the changes in relative displacements were consistent, indicating a smooth system transition. At 11:00, the transport system underwent a 90° rotation on the site plane, changing from occupying both directions to a single direction lane. Due to the influence of the single-direction lane’s cross slope, significant relative displacement changes occurred at various monitoring points. The monitoring data and on-site observations were used for timely correction to adjust the spatial orientation. In the subsequent transportation in the single-direction lane, the cut bridge body tilted towards the N1–N3 side, while the relative displacement at point N4 remained minimal. At 12:00, the cut bridge body was moved to the temporary storage area smoothly, and the relative displacements remained within a controllable range, effectively ensuring the safety of the transportation.

**Figure 23:**
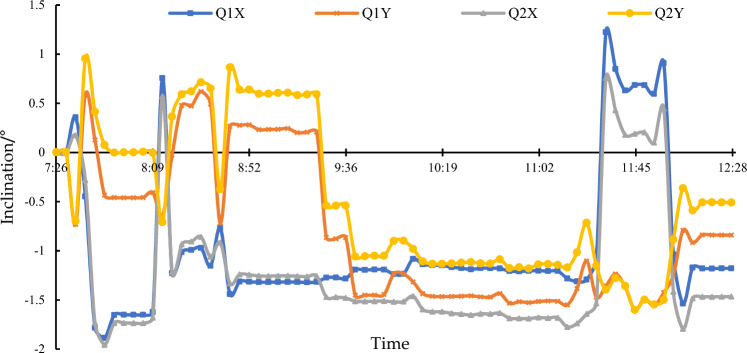
Monitoring of the inclination of the steel columns of the supporting brackets.

As the steel columns of the supporting brackets suffering the weight of the cut bridge body had relatively great lengths, their stability became a critical factor affecting construction safety. The verticality of the supports was influenced by two main factors: the precision of the production of the steel columns and the stress distribution in the steel columns. Low precision in production or uneven stress distribution may cause the steel columns to buckle. Therefore, the verticality of the steel columns was monitored in real-time, as shown in Fig. 23. High-precision inclinometers were placed on the steel columns in both X and Y directions, with a measurement range of ± 3°.

During the arch leg chain saw cutting (7:00 to 10:00), the load on the cut bridge body gradually transferred from the arch legs to the steel columns. The inclination of the steel columns remained within the range of ± 1° and showed consistent changes. From 10:00 to 11:00, the inclination of the steel columns changed significantly, approximately around 1.5°, mainly due to the posture changes of the transport system on-site. At the same time, based on the monitoring data and on-site observations, timely corrections were made to adjust the spatial orientation of the cut bridge body, ensuring that the inclination of the steel columns during the subsequent movement remained within a controllable range. According to the monitoring data, from 11:00 to 12:00 during the movement, the inclination of the steel columns tended to stabilize. The X and Y direction changes of monitoring points 1 and 2 were consistent and within the controllable range.

## Conclusions

A rapid demolition method of symmetrical rigid frame arch bridge crossing over an expressway was developed in this study based on SPMT, aiming to address the problems of low efficiency, high construction risks, and significant traffic impacts associated with conventional bridge demolition method during expressway expansion and reconstruction projects. Importantly, a monitoring system during the whole process was developed to ensure the demolition safety. An application of demolishing a symmetrical rigid frame arch bridge crossing over an expressway within 5 h was presented, which was a remarkable project in practice. The following conclusions can be drawn:

1. The derived calculation formulae for the reaction forces of supporting brackets, as well as driving force and stability of SPMTs could provide theoretical basis for the arrangement of SPMT. By applying the calculated support reaction forces, the movement of cut bridge was smooth and the inclination of columns in the brackets was always between ± 3° during the demolition process.
2. A monitoring system consisting of sensing devices, IoT cloud platform, processing platform was implemented. Timely feedback and on-site adjustments were made to ensure the safety and controllability of the rapid demolition process.

## Data Availability

The datasets used and/or analyzed during the current study are available from the corresponding author on reasonable request.
